# Effectiveness of Virtual Reality Interventions for Perioperative Anxiety in Adults: A Systemic Review With Meta‐Analysis

**DOI:** 10.1111/jocn.17806

**Published:** 2025-05-23

**Authors:** Salihah Asiri, Jane Currie, Jed Duff, Michelle Guilhermino

**Affiliations:** ^1^ School of Nursing Umm Al‐Qura University Makkah Saudi Arabia; ^2^ School of Nursing Faculty of Health, Queensland University of Technology Brisbane Queensland Australia; ^3^ Australian College of Perioperative Nurses (ACORN) Adelaide South Australia Australia; ^4^ Department of Perioperative Services Royal Brisbane & Women's Hospital Brisbane Queensland Australia; ^5^ School of Nursing and Midwifery College of Health, Medicine and Wellbeing, University of Newcastle Kelvin Grove Queensland Australia

**Keywords:** adult, anxiety, perioperative, preoperative, stress, surgery, virtual reality

## Abstract

**Background:**

Surgery often causes anxiety in adults due to various factors, including fear of anaesthesia and loss of independence. This anxiety can lead to higher anaesthesia requirements and more postoperative complications. Virtual Reality (VR) is increasingly used as a non‐pharmacological intervention to decrease perioperative anxiety. This systematic review and meta‐analysis aim to evaluate the effectiveness of VR to decrease perioperative anxiety in adult patients undergoing elective surgery.

**Design:**

A systematic review was conducted according to the Joanna Briggs Institute (JBI) method for systematic reviews of effectiveness. This paper complies with the Preferred Reporting Items for Systematic Reviews and Meta‐analyses (PRISMA) statement.

**Methods:**

Four electronic databases were searched from inception to July 2024. Inclusion criteria were experimental and quasi‐experimental studies using VR preoperatively to reduce perioperative anxiety as the primary outcome, with no language or date restrictions. Two independent reviewers screened and critically appraised the studies using the JBI appraisal tool. Randomised controlled trials (RCTs) were synthesised, and data were pooled in a statistical meta‐analysis. A random effects meta‐analysis was used due to heterogeneity. For studies that could not be meta‐analysed, a narrative synthesis was performed.

**Results:**

11 studies met the inclusion criteria, including eight RCTs and three quasi‐experimental studies. Overall, the majority of studies demonstrated that VR effectively reduced anxiety and increased patient satisfaction. The meta‐analysis revealed a small, non‐significant reduction in preoperative anxiety, favouring VR (Standardised Mean Difference − 0.29, 95% Confidence Interval − 0.83 to 0.24), with moderate heterogeneity (*I* ^2^ = 47%). However, findings regarding secondary outcomes including VR side effects, stress levels, preparedness levels, patients' satisfaction and length of hospital stay were inconsistent.

**Conclusions:**

The evidence is limited to support the use of VR in reducing perioperative anxiety among adults. Larger sample sizes, high‐quality RCTs and standardised anxiety measures are required to determine the effectiveness of VR on perioperative anxiety.

**Trial Registration:**

PROSPERO CRD42020158529 https://www.crd.york.ac.uk/prospero/display_record.php?RecordID=158529.

**Patient or Public Contribution:**

No patient or public contribution was required to design or undertake this review.

**Summary**

- What is already known?
  ○ Preoperative anxiety is common in adult surgical patients and can affect anaesthesia requirements and postoperative complications.
  ○ Virtual Reality (VR) is being used as a distraction and education tool to reduce perioperative anxiety.
  ○ Previous studies have shown mixed results on the effectiveness of VR in reducing anxiety among adult patients.
- What this paper adds?
  ○ This systematic review and meta‐analysis contribute to the understanding of VR's potential in reducing preoperative anxiety in adults.
  ○ VR shows promise in alleviating anxiety and increasing patient satisfaction, though statistical significance was not reached.
  ○ Larger, high‐quality RCTs are needed to establish conclusive evidence on VR's impact on perioperative anxiety.

## 1 Introduction

Globally, more than 312 million people are admitted for surgery every year (Weiser et al. 2015). The perioperative period, spanning preoperative, intraoperative and postoperative phases, can be an extremely stressful experience that elects specific emotional, cognitive, or physiological responses in surgical patients (Grupe and Nitschke 2013; McEwen and Karatsoreos 2020). This includes feelings of anxiety, fear and uncertainty, facing cognitive disruptions and undergoing physiological changes such as increased heart rate and elevated blood pressure (Grupe and Nitschke 2013; McEwen and Karatsoreos 2020). Anxiety during the perioperative period can stem from various causes, including apprehension about potential complications during the procedure, the unfamiliarity of the operating theatre environment, concerns about the extent and duration of postoperative disability, fear of losing control under general anaesthesia and anxiety about waking up and experiencing pain or discomfort during or after the surgery (Friedrich et al. 2022).

Perioperative anxiety can be described as a vague, uneasy feeling, where the source is often nonspecific and unknown to the individual (Klopfenstein et al. 2000). The prevalence of preoperative anxiety has been estimated by a number of studies worldwide, in both developed and developing countries. According to these studies, the prevalence of preoperative anxiety ranges from 21% to 80%, with a global pooled prevalence of 48% (Abate et al. 2020). There is a lack of available data regarding the prevalence of intraoperative and postoperative anxiety. A high level of preoperative anxiety has been associated with disruption in intraoperative procedures, presenting challenges during intubation and negatively impacting postoperative recovery by increasing pain perception, increasing the risk of postoperative nausea and vomiting, delaying wound healing and longer recovery (Bradshaw et al. 2016; Chieng et al. 2014; Laufenberg‐Feldmann et al. 2019; Li et al. 2021). High preoperative anxiety can also result in receiving a larger dose of anaesthesia, an increased risk of postoperative complications and lower levels of patient satisfaction with perioperative care (Celik and Edipoglu 2018; Kang et al. 2020; Stamenkovic et al. 2018).

Preoperative anxiety has been managed with various pharmacological and non‐pharmacological strategies. Pharmacological interventions include medications such as fentanyl, midazolam, morphine and ketamine (Jeon et al. 2018; Thong et al. 2021; Xiong et al. 2022). Side effects can occur from these medicines, such as breathing difficulties, drowsiness and interactions with other anaesthesia drugs (Curran and Lally 1999; Garcia et al. 2021). As a result, non‐pharmacological interventions, including music, massage, aromatherapy and acupuncture, guided imagery interventions and Virtual reality (VR) are becoming increasingly popular (Alvarez‐Garcia and Yaban 2020; Guo et al. 2020; Tola et al. 2021).

With advancement in technology, VR is gaining attention as an alternative or adjunct to medication for reducing perioperative anxiety. VR has been defined as a computer‐generated display that gives users the illusion of being in an environment other than their actual one and allows them to interact with it (Schroeder 1996). This technology is a major area of interest within the field of perioperative anxiety due to its minimal invasiveness and limited side effects (Kim and Lee 2019). VR involves multimodal stimulation, such as visual, auditory, tactile and even olfactory, to give users an unparalleled sense of actual immersion in a virtual world (Schroeder 1996). Different VR technologies can be sorted into two types: non‐immersive VR and immersive VR. In non‐immersive VR, users engage with a 3D environment using a regular desktop setup including a monitor, keyboard and mouse, while immersive VR offers users a more complete experience, making them feel surrounded by a virtual world. This is achieved through tools like a Head‐Mounted Display or multiscreen stereoscopic projection displays (Liu et al. 2023).

In a perioperative context, VR technology can be used as a distraction tool to divert patients' attention from their concerns about their upcoming surgery and can be used to provide preoperative education (Shepherd et al. 2022; Simonetti et al. 2022). For example, VR intervention has been used among the paediatric population to reduce preoperative anxiety with promising results according to the current systematic review and meta‐analysis (Pestana‐Santos et al. 2021; Simonetti et al. 2022). However, there have been few studies on the effect of VR on perioperative anxiety in adults, and therefore, evidence of the effectiveness of VR on perioperative anxiety among surgical patients is still in demand. Additionally, there is a need to evaluate the effects of VR on other perioperative outcomes, such as stress levels, patient preparedness, postoperative pain, satisfaction and length of hospital stay. A preliminary search of PROSPERO, MEDLINE, the Cochrane Database of Systematic Reviews and the JBI Database of Systematic Reviews and Implementation Reports was conducted, and no current or underway systematic reviews on the topic were found.

### 1.1 Objective

This systematic review and meta‐analysis aims to evaluate the effectiveness of using VR technology for perioperative anxiety among adults undergoing elective surgery.

### 1.2 Review Questions

1. Does VR administer preoperatively affect perioperative anxiety?
2. Does VR administer preoperatively affect perioperative outcomes such as stress levels, patient preparedness, postoperative pain, satisfaction and length of hospital stay?

## 2 Methods

This systematic review has been conducted in accordance with the Joanna Briggs Institute method for systematic reviews of effectiveness evidence (Peters et al. 2020). It is reported in accordance with the Preferred Reporting Items for Systematic Review and Meta‐analyses (PRISMA) guideline (Page et al. 2021; Appendix S1).

### 2.1 Protocol and Registration

The systematic review protocol was developed and registered by the International Prospective Register of Systematic Reviews (PROSPERO; CRD42020158529) in April 2020.

### 2.2 Eligibility Criteria

#### 2.2.1 Population

This review included adult patients aged 18 years who underwent elective surgery regardless of surgical type. Elective surgery is a surgical procedure that can be scheduled in advance and is not an emergency.

#### 2.2.2 Intervention

The review included VR interventions (immersive and non‐immersive) administered preoperatively for any purpose such as education, relaxation, or distraction.

#### 2.2.3 Comparison

The intervention was compared to usual/standard care, other non‐pharmacological interventions, either technological (e.g., mobile applications, educational videos) or non‐technological interventions. Pharmacological interventions were not included unless used as a part of routine preoperative care.

#### 2.2.4 Outcome

The primary outcome was perioperative anxiety. Secondary outcomes included VR side effects, stress levels, preparedness levels (referring to the patient's readiness for surgery) postoperative pain, patient satisfaction and length of hospital stay. These outcomes were measured by subjective or objective methods.

#### 2.2.5 Study Design

Eligible studies were experimental and quasi‐experimental designs including randomised controlled trials, non‐randomised controlled trials, before and after studies and interrupted time‐series studies. The inclusion criterion based on PICOS format is detailed in Table 1.

**TABLE 1:** Inclusion criteria based on PICOS format.

Review question	Inclusion criteria
Population	Adult patients aged 18 years, underwent elective surgery
Intervention	Virtual Reality during preoperative period for any purposes such as education, relaxation, or distraction
Comparator	Usual/standard care or other non‐pharmacological interventions
Outcome	Perioperative anxiety, VR side effects, stress levels, preparedness levels, postoperative pain, patient satisfaction and length of hospital stay
Study design	Experimental and quasi‐experimental study designs including randomised controlled trials, non‐randomised controlled trials, before and after studies and interrupted time‐series studies

#### 2.2.6 Exclusion Criteria

Studies were excluded if they involved patients under 18 years old who underwent emergency surgery. The VR intervention that was administered intraoperatively or postoperatively was also excluded. Studies with non‐experimental designs, such as observational studies or case reports, were also not included.

### 2.3 Search Strategy

A comprehensive systematic search strategy was developed in consultation with an expert research librarian. The search was conducted in four electronic databases: Cumulative Index to Nursing and Allied Health Literature (CINAHL), medical Literature Analysis and Retrieval System Online (MEDLINE), Excerpta Medica Database (Embase) and Psychological Information Database (PsycINFO) to seek published literature with no language and time limit from the inception of each database to July 2024. To check for any further relevant articles, we performed a targeted search in google scholar and searched trial registries for ongoing and unpublished trials. In addition, we searched the grey literature including conference proceedings and dissertations within the last 5 years. Finally, to check for any further relevant articles, a manual search of the reference lists of the identified studies was performed. Although we were unable to locate any unpublished studies related to our research question, we took steps to acknowledge the potential impact of publication bias on our review and highlighted the importance of considering all available literature sources when conducting a comprehensive review. Search terms are illustrated in Table 2

**TABLE 2:** Search strategy.

Database	Search statements	Number of results
CINAHL	((“Virtual Reality Exposure Therapy” OR “Virtual Reality Immersion Therapy” OR “Virtual Reality Therapy” OR “Virtual Reality Therapy*” OR “Computer Simulation” OR “Virtual Reality” OR “Virtual Reality Technology” OR “Human‐Computer Interaction”) AND (Surgery OR “Elective Surgery” OR “Scheduled Surgery” OR “Perioperative Care” OR “Surgical Procedures” OR Operative OR “Preoperative Care” OR Surgical OR Preop* OR Periop* OR Preoperat*) AND (Anxiety OR Anxiet* OR Nervousness OR Fear OR Panic OR Distress OR “Emotional Stress” OR “Psychological Stress” OR Anxious OR “Feel* of Apprehens*” OR “Feel* of Dread” OR “Feel* of Worry” OR “Feel* of Worried” OR “Feel* of Terror”))	104 Limit to adults = 32
MEDLINE	((“virtual reality exposure therapy” OR “virtual reality immersion therapy” OR “virtual reality therapy” OR “virtual reality therapy*” OR “computer simulation” OR “virtual reality” OR “virtual reality technology” OR “human‐computer interaction”) AND (surgery OR “elective surgical procedures” OR “scheduled surgical procedures” OR “perioperative care” OR “operative surgical procedures” OR “preoperative care” OR surgical OR preop* OR periop* OR preoperat*) AND (anxiety OR anxiet* OR nervousness OR fear OR panic OR distress OR “emotional stress” OR “psychological stress” OR anxious OR “feelings of apprehension” OR “feelings of dread” OR “feelings of worry” OR “feelings of terror”))	316 Limit to adults = 93
PsycINFO	((“virtual reality exposure therapy” OR “virtual reality immersion therapy” OR “virtual reality therapy” OR “virtual reality therapy*” OR “computer simulation” OR “virtual reality” OR “virtual reality technology” OR “human‐computer interaction”) AND (surgery OR “elective surgery” OR “scheduled surgery” OR “perioperative care” OR “surgical procedures” OR operative OR “preoperative care” OR surgical OR preop* OR periop* OR preoperat*) AND (anxiety OR anxiet* OR nervousness OR fear OR panic OR distress OR “emotional stress” OR “psychological stress” OR anxious OR “feel* of apprehens*” OR “feel* of dread” OR “feel* of worry” OR “feel* of worried” OR “feel* of terror”))	80 Limit to adults = 9
EMBASE	((‘virtual reality exposure therapy’/exp. OR ‘virtual reality exposure therapy’ OR ‘virtual reality immersion therapy’/exp. OR ‘virtual reality immersion therapy’ OR ‘virtual reality therapy’ OR ‘virtual reality therapy*’ OR ‘computer simulation’/exp. OR ‘computer simulation’ OR ‘virtual reality’/exp. OR ‘virtual reality’ OR ‘virtual reality technology’ OR ‘human‐computer interaction’/exp. OR ‘human‐computer interaction’) AND (‘surgery’/exp. OR surgery OR ‘elective surgery’/exp. OR ‘elective surgery’ OR ‘schedule surgery’ OR ‘perioperative care’/exp. OR ‘perioperative care’ OR ‘surgical procedures’ OR operative OR ‘preoperative care’/exp. OR ‘preoperative care’ OR surgical OR preop*periop* OR preoperat*) AND (‘anxiety’/exp. OR anxiety OR anxiet* OR ‘nervousness’/exp. OR nervousness OR ‘fear’/exp. OR fear OR ‘panic’/exp. OR panic OR ‘distress’/exp. OR distress OR ‘emotional stress’/exp. OR ‘emotional stress’ OR ‘psychological stress’/exp. OR ‘psychological stress’ OR anxious OR ‘feel* of apprehens*’ OR ‘feel* of dread’ OR ‘feel* of worry’ OR ‘feel* of worried’ OR ‘feel* of terror’))	607 Limit to adults = 282

### 2.4 Study Selection

Identified studies were merged and imported into EndNote X9 software, and any duplicates were removed (The EndNote Team 2013). Two independent reviewers (SA and JD) screened the titles and abstracts to assess eligibility based on the inclusion criteria. Following the completion of the title and abstract screening stage, the full text of the selected citations was then assessed in detail against the inclusion criteria by the same two independent reviewers (SA and JD); disagreements were referred to a third member (MG) of the research team for resolution.

### 2.5 Quality Assessment

Included studies were critically appraised by two independent reviewers (SA and MG) using the standardised critical appraisal instruments from the Joanna Briggs Institute for RCTs (JBI Critical Appraisal Checklist For Randomised Controlled Trials Appendix S2) and quasi‐experimental studies (JBI Critical Appraisal Checklist For Quasi‐Experimental Studies Appendix S2) (Tufanaru et al. 2017). These tools help to evaluate the methodological quality of a study and assess how thoroughly it has addressed potential bias in its design, conduct and analysis (Tufanaru et al. 2017). Any disagreements that arose were resolved through discussion with a third reviewer (JD). Each item was scored (1 for ‘yes,’ or 0 for ‘no’ or ‘unclear’), and the sum for each study was transformed to a percentage. Items marked as not applicable ‘N/A’ were not counted toward the final score and were excluded from the percentage calculation. Most studies were given an overall rating of high quality (more than 80%) by the authors. According to the JBI essential assessment method guidance, quality can be classified into three levels: high (> 80%), moderate (50%–80%) and low (less than 50%) (Tufanaru et al. 2020).

### 2.6 Data Extraction

Data were extracted from included studies by two independent reviewers (SA and MG) using a customised data extraction instrument that captured the author (year), study population, study design, setting, type of surgery, VR intervention (moment of VR, VR equipment and VR treatment description, duration and purpose), standard care, outcome measures and main findings. All necessary data for the included studies in the review were available in the published articles. Therefore, no additional data were requested from the authors. EndNote X9 software. Any disagreements that arose between the reviewers were resolved through discussion with a third reviewer (JD). The extracted data are presented in Table 3.

**TABLE 3:** Data extraction of included studies.

#	Author	Study design/setting	Population sample size and type of surgery	Aim/objectives	Intervention vs. control	Measurement tools	Study outcomes	Key findings	
	Year								
	Country								
1	Baytar and Bollucuoğlu (2021) Turkey	Design: prospective, observational cohort trial. Setting: Zonguldak Bülent Ecevit University Medicine Faculty Hospital	Sample size *N* = 40 Population: age 18–65 Surgery type: Elective septorhinoplasty	To evaluate the effect of VR video on preoperative anxiety, hemodynamic parameters and patient satisfaction	VR Duration: 15‐min VR video Content & Purpose: Natural scenes and sounds from nature for relaxation VR equipment: VR headset (Samsung Gear), fitted with a smart phone (Samsung Note 7 Edge) Moment of VR: preoperative waiting area	Primary outcomes: preoperative anxiety. hemodynamic parameter (BP, HR, SpO2) Other outcomes: patient satisfaction	Primary outcomes: 1. State Anxiety Inventory (STAI‐S) was obtained before and after the VR. 2. hemodynamic parameter were recorded before the VR and at 5, 10 and 15 min during the VR session. Other outcomes: satisfaction with the procedure (very satisfied, satisfied, undecided, unsatisfied), VR complication (headache, dizziness, nausea) and whether they would want this VR application again in case of a future surgery	STAI scores decreased significantly from 40.5 to 34 after VR intervention (*p* < 0.001, *d* = 1.28), with a large effect on preoperative anxiety. Significant differences were found in HR (*p* < 0.001), SBP (*p* < 0.001) and DBP (*p* = 0.001) before VR and 5‐, 10‐ and 15‐min during VR. No significant difference in SpO2 (*p* = 0.531). 90% of patients were satisfied with VR and would reuse it for future surgeries. Only one patient reported mild dizziness	
2	Bekelis et al. (2017b) United Kingdom (UK)	Design: Single centre RCT Setting: Tertiary referral centre	Sample size *N* = 127 Intervention: *N* = 64 Control: *N* = 63 Stratified by surgery type Population: adult going for surgery for the first‐time Surgery type cranial &spinal surgeries	Investigate the effect of educational VR preoperatively on Patient‐reported outcomes	VR Duration: 5 min (patients could watch the video as many times they wanted) Content &Purpose: Video aiming to describe the preoperative and postoperative experience for the day of the surgery for educational purpose Moment of VR: not reported VR equipment: VR goggles—Oculus VR & earplugs. Control Duration: not reported Content & Purpose: Standard care audio‐visual descriptions of the preoperative experiences & physician explained to patients what the preoperative experience would entail for education Moment of control activity: not reported	Primary outcomes: Evaluation du Vecu de I'Anesthesie Generale (EVAN‐G) score measured in the postoperative period within 24 h & Amsterdam Preoperative Anxiety and Information (APAIS) score was obtained Secondary outcomes: Visual Analog Scales (VAS) measuring patient pain and satisfaction pain, and satisfaction were obtained preoperatively on the day of surgery and postoperatively within 24 h of the surgery For spine patients, pain scale was obtained 30 days postoperatively	Primary outcomes: 1. Perioperative patient satisfaction 2. Preoperative anxiety Secondary outcomes: 1. Pre and postoperative stress level 2. Pre and postoperative satisfaction level 3. Pre and postoperative preparedness level 4. Pre and postoperative pain levels	EVAN‐G score was significantly higher after the VR (84.3) (SD, 6.4) than after the standard procedure 64.3 (SD, 11.7). (Note: higher scores representing higher satisfaction) APAIS score was significantly higher after VR (90.7) than after the standard procedure (60.8), (Note: higher scores representing lower anxiety). VR led to significantly: lower preoperative stress (difference, −41.7; 95% CI, −33.1 to −50.2) and higher preparedness s (difference, 32.4; 95% CI, 24.9–39.8) and higher satisfaction scores (difference, 26.4; 95% CI, 20.1–32.6). No effect of VR on average preoperative and postoperative pain	
3	Ganry et al. (2018) France	Design: Prospective single‐blind pilot study. Setting: outpatient department at a teaching hospital	Sample size *N* = 20 Population: Adults ≥ 18 years. with Amsterdam Preoperative Anxiety and Information (APAIS) score of 11 or above Surgery type: Skin cancer	Determine whether a VR presenting natural scenes preoperatively could reduce patients' anxiety	VR Duration: 5‐min VR video Content & Purpose: Natural scenes and sounds from nature for relaxation. VR equipment: VR glasses (Oculus), an audio headset (Oculus Rift) connected to a LDLC computer Moment of VR: preoperative waiting area	Primary outcomes: Visual Analog Scale (VAS) measuring level of anxiety was obtained before and after the VR. Salivary cortisol was obtained right before the VR and after the VR & Heart coherence score was obtained over 3 min before and after VR	Primary outcome: 1. Stress levels 2. Salivary cortisol levels 3. Heart coherence scores.	VAS score significantly reduced from 3.3/10 to 2.85/10 after VR (*p* < 0.009). Salivary cortisol level significantly reduced after VR (concentration before 14.55 and 12.86 after < 0.04). Heart coherence scores were unchanged after VR (average score 50.6 before and 46.6 after VR, *p* = 0.056)	
4	Hendricks et al. (2020) United states of America (USA)	Design: pilot RCT Setting: Surgical clinic	Sample size: *N* = 20 Intervention: *N* = 10 Control: *N* = 10 Population: adults going to a sternotomy for the first‐time. Surgery type: Cardiac surgery	Investigate if immersed VR game can reduce patient perceptions of anxiety compared to non‐immersive tablet‐based game in adults' cardiac patients	VR Duration: 20 min Content &Purpose of VR: Play VR game model titled “Bear Blast” for distraction Moment of VR: preoperative room while waiting to transfer to preoperative waiting area VR equipment: Samsung Gear Oculus and audio headset fitted with Samsung Galaxy S7. Control Duration: not provided Content &Purpose: Tablet‐based game with audio‐visual‐tactile simulation (Candy Crush) for distraction Moment of control activity: preoperative room while waiting to transfer to preoperative waiting area Equipment: tablet	Primary outcomes: State–Trait Anxiety Inventory (STAI) was obtained before and after intervention Secondary outcomes: medical chart & electronic medical record was reviewed after the intervention	Primary outcomes: 1. Preoperative anxiety Secondary outcomes: 1. Difficulty of intubation 2. Postoperative nausea 3. Length of hospital stay. 4. preoperative anxiolytic administration usage(as‐needed)	VR users experienced significant reductions in feeling tense and strained(*d* = 2.2) and significant improvements in feeling calm(*d* = 2.1) compared with tablet control (*p* < 0.05). Difficulty of intubation, postoperative nausea and length of stay were not significantly different for each intervention. Use of anxiolytic medication was slightly lower in the VR group compared to tablet control between the end of the VR session and induction for surgery (*p* = 0.08)	
5	Kapikiran et al. (2022) Turkey	Design: Pre‐test and post‐test experimental Setting: The organ transplantation unit of a university hospital in Turkey	Sample size *N* = 120 Interventions: *N* = 60 Control: *N* = 60 Population: Adults 18–60 years Surgery type: Transplant surgery	To examine the effect of patient information provided with HMD before transplant surgery on patient satisfaction and anxiety	VR Duration: 34‐min VR video Content & Purpose: video on various pre‐ and post‐operative topics for education VR equipment: HMD and no more details Moment of VR: 24 to 72 h before their surgery. Control Duration: not mentioned Content & Purpose: Verbally information provided by a clinical nurse topic for education Moment of control activity: not mentioned	Primary outcomes: 1. anxiety specific to surgery questionnaire (ASSQ) ranging from 0 to 50 measuring level of anxiety was obtained two time points: 24–36 h before surgery, 8–12 h before surgery, surgery. 2. the Newcastle Satisfaction with Nursing Care Scale (SNCS) ranging from 0 to 100 measuring level of satisfaction was obtained two time points: 24–36 h before surgery and 48–72 h after surgery. 3. length of hospital stay	Primary outcomes: 1. anxiety levels 2. satisfaction levels 3. length of hospital stay	Among the VR group, there was a significant difference in mean pertest and post‐test in: ASSQ scores (*p* = 0.001, *t* = 2.311) and SNCS scores (*p* = 0.000, *t* = 1.214). significant, moderately negative relationship between mean ASSQ and SNCS scores (*r* = −0.613, *p* = 0.000). significantly shorter hospitalisation compared to the control group (*χ* ^2^ = 7.51, *p* = 0.000, *F* = 2.154). No statistically significant difference in mean pertest and post‐test ASSQ scores of the control group (*p* = 0.807, *t* = 1.784)	
6	Keshvari et al. (2021) Iran	Design: single‐centre RCT Setting: Central cardiac hospital	Sample size *N* = 80 Intervention: *N* = 40 Control: *N* = 40 Population: mean age 57.5 Surgery type: Coronary angiography	To investigate the effect of VR on anxiety before coronary angiography	VR Duration: 5‐min VR video Content & Purpose: Natural scenes and sounds from nature for relaxation. VR equipment: VR headset (Remix company), fitted with a phone (Huawei) Moment of VR: 10 min before patients being taking to the operating room. Control Duration: not reported Content/Purpose: not reported Moment of control activity: 10 min before patients being taking to the operating room	Primary outcomes: 1. State–Trait Anxiety Inventory (STAI) short form (ranging from 6 to 24) was obtained before and after intervention 2. BP, HR and RR were obtained before and after the VR. BP was measured using an Easy Life hand‐held sphygmomanometer in a supine, right‐hand position and HR and RR were obtained by digital watch made by CASIO was obtained before and after intervention	Primary outcomes: 1. Preoperative anxiety 2. vital signs (heart rate, respiratory rate and blood pressure)	71.25% were male. Significant decrease in anxiety score among VR compared to control (*p* = 0.002). Significant difference in the mean HR among VR compared to control (*p* = 0.035)	
7	Robertson et al. (2017) Australia	Design: single‐centre RCT Setting: private hospital in Perth	Sample size *N* = 60 Interventions: 1. VR group *N* = 20 2. iPad group *N* = 20 Control: *N* = 20 Population: age Adults ≥ 18 years. Surgery type: Arthroscopic knee surgery	To evaluate the feasibility and clinical potential of immersive VR in reducing anxiety in the preoperative setting.	VR Duration: 9‐min VR video Content & Purpose: Beach for relaxation. VR equipment: VR headset (Samsung Gear VR Headset fitted with Samsung Note 4 mobile phone) + a pair of QuietComfort25 Acoustic Noise Cancelling Headphones Moment of VR: preoperative waiting area. iPad Duration: 9‐min Content & Purpose: Beach for relaxation Moment of iPad: preoperative waiting area. Control Duration: not reported Content & Purpose: not mentioned Moment of control activity: preoperative waiting area	Primary outcome: Anxiety scores (Hospital Anxiety and Depression Scale), ranging from 0 to 21, two time points: before and after the intervention. Objective measurements of anxiety: ‐Galvanic Skin Response (GSR), recorded over the entire nine‐minute waiting period. ‐HR, BP two time points: before and after the intervention	Primary outcome: 1. Preoperative anxiety	HADS: Standard care group: Mean decrease of −0.32 (SD = 1.63) iPad group: Mean decrease of −1.47 (SD = 2.63) VR group: Mean decrease of −1.6 (SD = 2.33) Marginal difference between standard and VR groups (t(37) = 1.99, *p* = 0.055, Cohen's *d* = 0.63) No significant difference between the VR and iPad groups (*t* < 1). HR: No difference between the standard and VR groups (*t* < 1) No difference between the iPad and VR groups (*t*(35) = 1.65, *p* = 0.108). GSR: Significant difference between standard and VR groups (*t*(36) = 2.04, *p* < 0.048) No significant difference between the iPad and VR groups (*t* < 1) BP: Systolic BP: No difference between the standard care and VR groups (*t* < 1) No difference between the iPad and VR groups (*t* < 1) Diastolic BP: No difference between the standard and VR groups (*t*(34) = −1.47, *p* = 0.152) Marginal difference between iPad and VR groups (*t*(33) = −2.04, *p* = 0.050, Cohen's *d* = 0.65) VR intervention was well tolerated by all participants, with no reports of motion sickness or discomfort	
8	Turrado et al. (2021) Spain	Design: single centre RCT Setting: Gastrointestinal surgery Department of a third‐level academic center in Barcelona	Sample size *N* = 126 Interventions: *N* = 58 Control: *N* = 68 Population: Adults Surgery type: Elective surgery for colorectal cancer	To evaluate the effectiveness of exposure to the entire perioperative environment through VR in decreasing the preoperative anxiety of patients with colorectal cancer compared to the standard care	VR Duration: 16 min and 34 s VR video Content & Purpose: realistic environment of the perioperative process and the participants could view all the phases of the perioperative process without interruption or select any of them specifically for education. VR equipment: Bluebee Genuine VR 3D Glasses, adaptable to any smartphone model Moment of VR: on the day of their surgery. Control No details provided	Primary outcome: 1. two scales were the State–Trait Anxiety Inventory Scale State (STAI‐S), ranging from 20 to 80, and the Hospital Anxiety and Depression Scale (HADS), ranging from 0 to 21, measuring level of preoperative anxiety was obtained two time points in VR group and one time point in control group on the day of their surgery	Primary outcomes: 1. preoperative anxiety levels Other outcomes: 1. length of hospital stay. 2. VR Side effect and complication rate without mentioned the evaluation tools that used	HADS and STAI‐S scores were significantly lower in VR group (*p* < 0.001). No adverse effects, except for one patient experiencing dizziness Overall complication rate was 5.55%, slightly lower in the VR group (5.17%) compared to non‐VR group (5.88%) Median hospital stay was 3.1 days (95% CI 2.3–4.8 days) and did not differ between VR and control groups	
9	Ugras et al. (2022) Turkey	Design: single‐centre RCT Setting: general surgery unit of a university hospital	Sample size *N* = 86 Intervention: *N* = 43 Control: *N* = 43 Population: age 18–65 Surgery type: Elective colorectal and abdominal wall surgery	To investigate the effects of a VR application on preoperative anxiety	VR Duration: 10‐min VR video Content & Purpose: Natural scenes and sounds from nature for relaxation VR equipment: VR headset (VR BOX 2), fitted with a phone (iPhone 7 Plus) Moment of VR: 10 min before patients being taking to the operating room. Control They did not receive any intervention	Primary outcome: psychological responses to preoperative anxiety, using Anxiety Specific to Surgery Questionnaire (ASSQ), ranging from 10 to 50 before and after the VR Secondary outcomes: physiological responses to preoperative anxiety (SBP, DBP, HR, RR and SpO2) were obtained before and after the VR	Primary outcomes: 1. Preoperative anxiety Secondary outcomes: SBP, DBP, HR, RR and SpO2	ASSQ score significantly reduced from mean 30.9 (SD = 6.8) to mean 25.1 (SD = 6.5) after VR (*p* < 0.001) Significantly increased from mean 29.0 (SD = 5.8) to mean 29.7 (SD = 6.2) after standard care *F* = 75.823 Statistically significant difference in the SBP, DBP, HR, RR and SpO2 values at the different time points between the two groups	
10	Vogt et al. (2021) Germany	Design: Prospectively double‐blinded RCT Setting: Clinic for Anaesthesiology, RWTH Aachen University Hospital	Sample size *N* = 80 Intervention: *N* = 40 Control: *N* = 40 Population: age Adults ≥ 18 years. Surgery type: Elective surgery with general anaesthesia except for thoracic surgery, neurosurgery and tumour surgery	To investigate whether a virtual operating room tour (VORT) before surgery can be used to ameliorate perioperative anxiety	VR Duration: 6‐min and 28 s VR video Content & Purpose: virtual tour of the operation room for education. VR equipment: VR headset (Oculus Go Standalone VR) Moment of VR: during Anaesthesia Interview. Control Duration: not reported Content & Purpose: standardised information sheet used by the anaesthetist for education Moment of control activity: during Anaesthesia Interview	Primary outcomes: state–trait operation anxiety inventory (STOA)was obtained before and after the surgery ‐Trait anxiety (STOA‐T, 20 items) one time point, before surgery ‐state anxiety (STOA‐S, 10 items) two time points, one before surgery and one within 48 h after surgery Secondary outcomes: evaluation the virtual operation tour using house designed questionnaire	Primary outcome: 1. Perioperative anxiety Secondary outcome: 1. evaluation the virtual operation tour	Before surgery: No significant differences in state anxiety between groups (VR mean 10.49, SD 4.61 vs. control mean 10.83, SD 4.09) in affective (*t* = −0.37, *p* = 0.74) and cognitive (VR mean 10.88, SD 4.32 vs. control mean 10.70, SD 4.38); (*t* = −0.18, *p* = 0.86) After surgery: Affective (*n* = 68); VORT: mean 8.53, SD 4.95 vs. STOPP: mean 7.91, SD 3.26; (*t* = 0.61; *p* = 0.55) and the cognitive (*n* = 71); VORT: mean 9.11, SD 4.19 vs. STOPP: mean 9.17, SD 4.00; (*t* = −0.05; *p* = 0.96). Most patients found the VORT helpful for preparation and would recommend it to others	
11	Yang et al. (2019) South Korea	Design: Single‐centre RCT Setting: hospital at Hanyang University	Sample size *N* = 48 Intervention: *N* = 24 Control: *N* = 24 Population: age 15–65 Surgery type: Elective arthroscopic knee surgery	Assess the effect of preoperative VR on patients' anxiety reduction	VR Duration: not reported Content & Purpose: Watch a 3D MRI result describing the knee anatomy and patients' lesion of interest for education Moment of VR: approximately 12 h before surgery VR equipment: Vive; HTC, New Taipei City, Taiwan. Control Duration: not reported Content & Purpose: Standard preoperative information was given by a single surgeon about their MRI through a picture‐archiving and communication system for education Equipment: π‐viewer v.5.0.8.1; Infinite, Seoul, Korea Moment of control activity: approximately 12 h before surgery	Primary outcomes: Amsterdam Preoperative Anxiety and Information (APAIS) score was obtained preoperatively on the day of the surgery Secondary outcomes: Visual Analog Scale (VAS) measuring patient pain, preparedness, satisfaction and stress were obtained twice preoperatively on the day of the surgery and postoperatively day 3	Primary outcomes: 1. Preoperative anxiety Secondary outcomes: 1. Stress level 2. Satisfaction level 3. preparedness level 4. Pain level	APAIS subscale (sum C) significantly better in VR group, median 4.0 (4.0–8.5) compared with control 8.0 (5.3–9.8) (*p* = 0.014) Preoperatively: Significantly better satisfaction in the VR group (*p* = 0.016) No significant difference between VR and standard group in measures of pain, preparedness and stress Postoperatively: Significantly better satisfaction in the VR group (*p* = 0.010) and significant stress level (*p* = 0.012) pain was not significantly different between the 2 groups	

### 2.7 Data Analysis and Synthesis

Studies were pooled with statistical meta‐analysis using RStudio(R Core Team 2020). Effect sizes were expressed as standardised final post‐intervention mean differences (for continuous data) and their 95% confidence intervals were calculated for analysis. For studies that reported median and interquartile range, conversion to mean and standard deviation was conducted (Luo et al. 2018; Wan et al. 2014). Heterogeneity was assessed statistically using the standard chi squared and I squared tests and further investigated through a leave one out analysis (Biggerstaff and Jackson 2008). Statistical analyses were performed using random‐effect models. The findings are presented and synthesised in a narrative format without meta‐analysis when statistical pooling was not possible. A funnel plot was generated to assess the potential publication bias in the included studies.

## 3 Results

### 3.1 Study Selection

**FIGURE 1:**
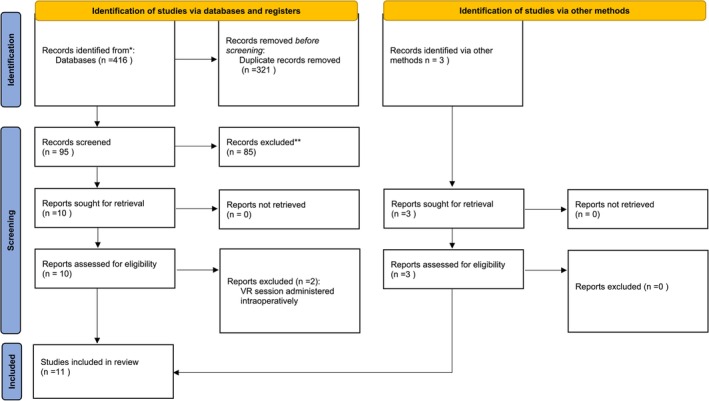
PRISMA 2020 flow diagram. *Consider, if feasible to do so, reporting the number of records identified from each database or register searched (rather than the total number across all databases/registers). **If automation tools were used, indicate how many records were excluded by a human and how many were excluded by automation tools.* * From: Page et al. (2021). For more information, visit: http://www.prisma‐statement.org/. [Colour figure can be viewed at wileyonlinelibrary.com]

A total of 419 studies were identified from various databases, and 321 duplicate records were removed. The remaining 98 records were screened, and 85 were excluded based on the inclusion and exclusion criteria. Full‐text review was conducted for 13 reports, and two were excluded. Eleven studies met the eligibility criteria and were included in the review. The study selection process is summarised in Figure 1, which follows the PRISMA guidelines for systematic reviews.

According to the JBI essential assessment method guidance, the quality assessment results categorise one study as low quality (Turrado et al. 2021), one as moderate quality (Hendricks et al. 2020), and nine as high‐quality studies (Baytar and Bollucuoğlu 2021; Bekelis et al. 2017a; Ganry et al. 2018; Kapikiran et al. 2022; Keshvari et al. 2021; Robertson et al. 2017; Ugras et al. 2022; Vogt et al. 2021; Yang et al. 2019). The quality assessment is presented in Table 4.

**TABLE 4:** Quality appraisal.

A. JBI critical appraisal checklist for randomised controlled trials								
c	Hendricks et al. (2020)	Bekelis et al. (2017b)	Yang et al. (2019)	Turrado et al. (2021)	Robertson et al. (2017)	Vogt et al. (2021)	Ugras et al. (2022)	Keshvari et al. (2021)
Was true randomisation used for assignment of participants to treatment groups?	Yes	Yes	Yes	Yes	Yes	Yes	Yes	Yes
Was allocation to treatment groups concealed?	Unclear	Yes	Unclear	Unclear	Yes	Yes	Yes	Yes
Were treatment groups similar at baseline?	Yes	Yes	Yes	Yes	Yes	Yes	Yes	Yes
Were participants blind to treatment assignment?	N/A	N/A	N/A	N/A	N/A	N/A	N/A	N/A
Were those delivering treatment blind to treatment assignment?	Unclear	Yes	Yes	Unclear	Unclear	Yes	Yes	Yes
Were outcome assessors blind to treatment assignment?	Unclear	Yes	Yes	Unclear	Unclear	Yes	Yes	Unclear
Were treatment groups treated identically other than the intervention of interest?	Yes	Yes	Yes	No	Yes	Yes	Yes	Yes
Was follow‐up complete, and if not, were strategies to address incomplete follow‐up utilised?	Yes	Yes	Yes	Yes	Yes	Yes	Yes	Yes
Were participants analysed in the groups to which they were randomised?	Yes	Yes	Yes	Yes	Yes	Yes	Yes	Yes
Were outcomes measured in the same way for treatment groups?	Yes	Yes	Yes	No	Yes	Yes	Yes	Yes
Were outcomes measured in a reliable way?	Yes	Yes	Yes	Unclear	Yes	Yes	Yes	Yes
Was appropriate statistical analysis used?	Yes	Yes	Yes	No	Yes	Yes	Yes	Yes
Was the trial design appropriate, and any deviations from the standard RCT design (individual randomisation, parallel groups) accounted for in the conduct and analysis of the trial	Yes	Yes	Yes	No	Yes	Yes	Yes	No
Score	77%	100%	92%	38%	85%	100%	100%	85%

B. JBI critical appraisal checklist for quasi‐experimental studies			
Question	Baytar and Bollucuoğlu (2021)	Ganry et al. (2018)	Kapikiran et al. (2022)
Is it clear in the study what is the ‘cause’ and what is the ‘effect’ (i.e., there is no confusion about which variable comes first)?	Yes	Yes	Yes
Were the participants included in any comparisons similar?	N/A	Yes	Yes
Were the participants included in any comparisons receiving similar treatment/care, other than the exposure or intervention of interest?	N/A	Yes	Yes
Was there a control group?	N/A	No	Yes
Were there multiple measurements of the outcome both pre and post intervention/exposure?	Yes	Yes	Yes
Was follow‐up complete and if not, were differences between groups in terms of their follow‐up adequately described and analysed?	Yes	N/A	Yes
Were the outcomes of participants included in any comparisons measured in the same way?	Yes	Yes	Yes
Were outcomes measured in a reliable way?	Yes	Yes	Unclear
Was appropriate statistical analysis used?	Yes	Yes	Yes
Score	100%	89%	89%

Abbreviation: N/A, not applicable.

### 3.2 Study Characteristics

This review included 11 studies, with sample sizes ranging from 20 to 126, totalling 567 participants. Of these studies, eight were RCTs (Bekelis et al. 2017a, 2017b; Hendricks et al. 2020; Keshvari et al. 2021; Robertson et al. 2017; Turrado et al. 2021; Ugras et al. 2022; Vogt et al. 2021; Yang et al. 2019), while three were quasi‐experimental studies (Baytar and Bollucuoğlu 2021; Ganry et al. 2018; Kapikiran et al. 2022).

#### 3.2.1 Population

These studies were conducted among adult surgical patients aged 18 years and older across various continents. Four studies were conducted in Europe, including the United Kingdom (Bekelis et al. 2017b), France (Ganry et al. 2018), Spain (Turrado et al. 2021) and Germany (Vogt et al. 2021). Five studies were conducted in Asia, including South Korea (Yang et al. 2019), three in Turkey (Baytar and Bollucuoğlu 2021; Kapikiran et al. 2022; Ugras et al. 2022) and Iran (Keshvari et al. 2021). Additionally, one study was conducted in Australia (Robertson et al. 2017), and another in the United States of America (Hendricks et al. 2020).

All of these studies were conducted between 2017 and 2022 and involved adults undergoing different types of surgeries, including cardiac surgery (Hendricks et al. 2020), cranial and spinal surgeries (Bekelis et al. 2017b), arthroscopic knee surgery (Robertson et al. 2017; Yang et al. 2019), skin cancer surgery (Ganry et al. 2018), colorectal and abdominal wall surgery (Ugras et al. 2022), coronary angiography (Keshvari et al. 2021), septorhinoplasty (Baytar and Bollucuoğlu 2021; Vogt et al. 2021), colorectal cancer surgery (Turrado et al. 2021) and transplant surgery (Kapikiran et al. 2022).

#### 3.2.2 Intervention

The applications of VR are in the form of distraction through games (Hendricks et al. 2020), distraction through watching and listening to calming content for relaxation (Baytar and Bollucuoğlu 2021; Ganry et al. 2018; Keshvari et al. 2021; Robertson et al. 2017; Ugras et al. 2022), and also VR is used for patient education through informative surgical content (Bekelis et al. 2017b; Kapikiran et al. 2022; Turrado et al. 2021; Vogt et al. 2021; Yang et al. 2019). The duration of VR interventions ranged from 5 to 34 min; however, one study did not report the duration of VR interventions (Yang et al. 2019).

The VR interventions were conducted in various settings, including the preoperative waiting area (Baytar and Bollucuoğlu 2021; Ganry et al. 2018; Hendricks et al. 2020; Keshvari et al. 2021; Robertson et al. 2017; Turrado et al. 2021; Ugras et al. 2022), around 12 h before surgery (Yang et al. 2019), 24–72 h before surgery (Kapikiran et al. 2022) and during the anaesthesia interview (Vogt et al. 2021). However, one study did not report when the VR intervention was administered (Bekelis et al. 2017b).

A range of hardware and software devices, including Samsung Gear, Oculus, Oculus glasses, Vive by HTC, VR BOX 2, Remix Headset and Bluebee Genuine VR 3D Glasses, were used for VR interventions. These VR devices were connected to different smartphones and computers.

#### 3.2.3 Comparator

The interventions in the control groups among RCTs were not consistent across the studies. Some studies used tablet‐based interventions (iPad) for both distraction purposes (Hendricks et al. 2020; Robertson et al. 2017), while others used audio‐visual descriptions (Bekelis et al. 2017a; Yang et al. 2019), standardised information sheets (Vogt et al. 2021), or verbal information (Kapikiran et al. 2022) for education purposes. However, some studies reported that standard preoperative care was provided among the control group without further explanation (Keshvari et al. 2021; Robertson et al. 2017; Turrado et al. 2021). In Ugras et al. (2022) study, the control group did not receive any intervention, and it remains unclear whether they received standard care. In Robertson et al. (2017), a three‐arm design was used, comparing VR versus iPad and VR versus standard care, while all other RCTs used a two‐arm design. Due to the observed clinical heterogeneity among the included studies, a decision was made to conduct a meta‐analysis solely on comparable groups, focusing on the outcomes of mean anxiety levels before and after the interventions.

### 3.3 Outcomes

#### 3.3.1 Primary Outcome

##### 3.3.1.1 Effects of VR on Perioperative Anxiety

###### 3.3.1.1.1 Study Overview

All included studies in this review primarily focused on measuring preoperative anxiety. There was only one study conducted by Vogt et al. (2021) that assessed both preoperative and postoperative anxiety. Different subjective assessment tools were used across the studies, including the State–Trait Anxiety Inventory (STAI) (Baytar and Bollucuoğlu 2021; Hendricks et al. 2020; Keshvari et al. 2021; Turrado et al. 2021), Amsterdam Preoperative Anxiety and Information Scale (APAIS) (Bekelis et al. 2017a; Yang et al. 2019), Hospital Anxiety and Depression Scale (HADS) (Robertson et al. 2017; Turrado et al. 2021), Anxiety Specific to Surgery Questionnaire (ASSQ) (Kapikiran et al. 2022; Ugras et al. 2022), State–Trait Operation Anxiety (STOA) (Vogt et al. 2021) and Visual Analogue Scale for Anxiety (VAS‐A) (Ganry et al. 2018).

In addition to subjective assessment tools, objective measurement tools were used in five studies to measure preoperative anxiety (Baytar and Bollucuoğlu 2021; Ganry et al. 2018; Keshvari et al. 2021; Robertson et al. 2017; Ugras et al. 2022). These objective measures included biochemical markers, such as salivary cortisol levels (Ganry et al. 2018) and galvanic skin response (GSR) (Robertson et al. 2017) and physiological parameters including heart coherence, systolic blood pressure, diastolic blood pressure, heart rate, respiratory rate and oxygen saturation (Ganry et al. 2018; Keshvari et al. 2021; Robertson et al. 2017; Ugras et al. 2022). A summary of the subjective and objective measurement tools used to assess perioperative anxiety in the included studies is presented in Table 5.

**TABLE 5:** Subjective and objective measurement tools used to assess perioperative anxiety.

Study author	Subjective measures	Objective measures
Hendricks et al. (2020)	STAI	Nil
Bekelis et al. (2017a)	APAIS	Nil
Yang et al. (2019)	APAIS	Nil
Ganry et al. (2018)	VAS‐A	Salivary cortisol levels and heart coherence scores
Ugras et al. (2022)	ASSQ	Hemodynamic (SBP, DBP, HR, RR and SpO2)
Keshvari et al. (2021)	STAI (short form)	Hemodynamic (BP, HR and RR)
Baytar and Bollucuoğlu (2021)	STAI	Hemodynamic (BP, HR and SpO2)
Vogt et al. (2021)	STOA	Nil
Robertson et al. (2017)	HADS	GSR and Hemodynamic parameters (SBP, DBP and HR)
Turrado et al. (2021)	STAI and HADS	Nil
Kapikiran et al. (2022)	ASSQ	Nil

Abbreviations: ASSQ, Anxiety Specific to Surgery Questionnaire; BP, blood pressure; GSR, galvanic skin response; HADS, Hospital Anxiety and Depression Scale; HR, heart rate; PAIS, Amsterdam Preoperative Anxiety and Information Scale; RR, respiratory rate; SpO2, peripheral oxygen saturation; STAI, Staaaaate–Trait Anxiety Inventory; STOA, State–Trait Operation Anxiety Inventory; VAS‐A, Visual Analog Scale for Anxiety.

The results related to the effectiveness of VR in reducing perioperative anxiety among the included studies have been based on whether the intervention was used for educational purposes or distraction purposes. Among the five studies that used VR for educational purposes, and based on subjective measurement tools (Bekelis et al. 2017a; Kapikiran et al. 2022; Turrado et al. 2021; Vogt et al. 2021; Yang et al. 2019), four studies showed a significant reduction of preoperative anxiety among the VR group (Bekelis et al. 2017a; Kapikiran et al. 2022; Turrado et al. 2021; Yang et al. 2019), while the remaining study conducted by Vogt et al. (2021) did not find a significant difference in preoperative anxiety levels between the VR and control groups (*t* = −0.37, *p* = 0.74); the same was observed for postoperative anxiety (*t* = 0.61; *p* = 0.55). The timing of the intervention differed in this study in that it was administered during the anaesthesia interview; however, the exact timing of the interview itself was not explicitly provided.

Among the six studies that used VR for distraction purposes and based on subjective measurement tools (Baytar and Bollucuoğlu 2021; Ganry et al. 2018; Hendricks et al. 2020; Keshvari et al. 2021; Robertson et al. 2017; Ugras et al. 2022), five studies showed a significant reduction in preoperative anxiety among the VR groups. The remaining study conducted by Robertson et al. (2017) using a three‐arm RCT showed a non‐significant improvement in the VR group compared to the control groups (*t* (37) = 1.99, *p* = 0.055, Cohen's *d* = 0.63). Interestingly, there was no significant difference observed between the VR and iPad groups (*t* < 1).

Five out of the six distraction VR studies used objective measures including biochemical markers and haemodynamic parameters (Baytar and Bollucuoğlu 2021; Ganry et al. 2018; Keshvari et al. 2021; Robertson et al. 2017; Ugras et al. 2022). For biochemical markers, Robertson et al. (2017) reported a significant difference in GSR between the control and VR groups (*t*(36) = 2.04, *p* < 0.048), indicating a greater reduction in GSR for the VR group. However, there was no significant difference in GSR between the iPad and VR groups (*t* < 1). Similarly, Ganry et al. (2018) reported a significant decrease in salivary cortisol levels from 14.55 to 12.86 (*p* < 0.04) after the VR session.

Regarding haemodynamic parameters, five out of six distraction studies measured the effects of VR on parameters such as heart coherence, heart rate, blood pressure, respiratory rate and peripheral oxygen saturation level (Baytar and Bollucuoğlu 2021; Ganry et al. 2018; Keshvari et al. 2021; Robertson et al. 2017; Ugras et al. 2022). The findings on haemodynamic measures were inconsistent and did not support the effectiveness of VR in altering these parameters. In most studies, significant reductions in HR were observed following VR interventions, suggesting a potential calming effect (Baytar and Bollucuoğlu 2021; Keshvari et al. 2021; Ugras et al. 2022).

**FIGURE 2:**
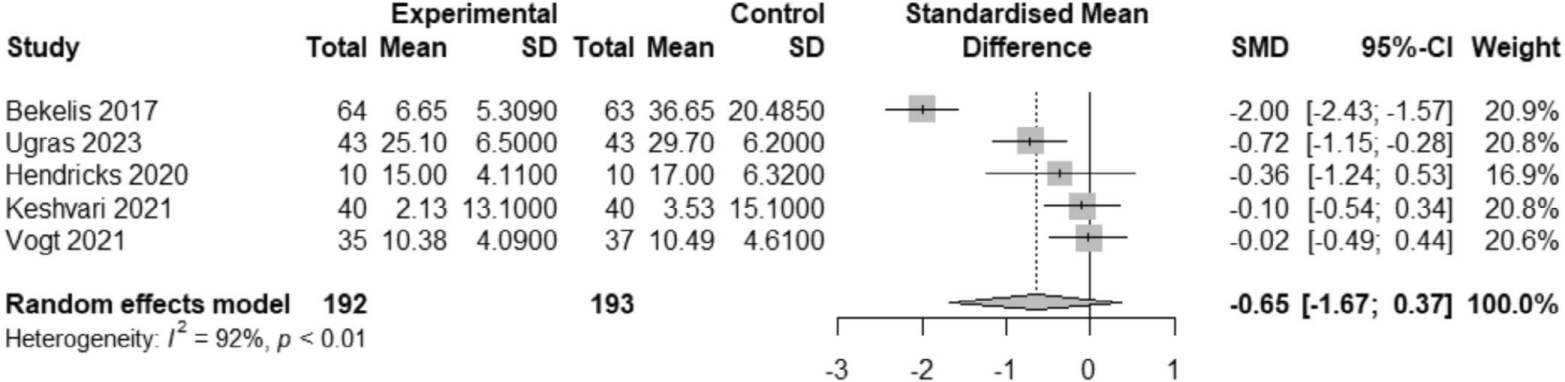
Forest plot of VR interventions compared with the control group for preoperative anxiety in adults.

The meta‐analysis was conducted with the five RCT studies (Bekelis et al. 2017a; Hendricks et al. 2020; Keshvari et al. 2021; Ugras et al. 2022; Vogt et al. 2021) and included a total of 385 adults' surgical patients who underwent elective surgery. As presented in Figure 2, the findings indicate a medium effect (SMD −0.65, 95% CI −1.67 to 0.37) reduction in preoperative anxiety from VR; however, the confidence interval includes 0, and thus, this result is not significant. Heterogeneity between the studies was above 90% (*I* ^2^ = 92%, *p* < 0.01), which is considered substantial (Fletcher 2007).

**FIGURE 3:**
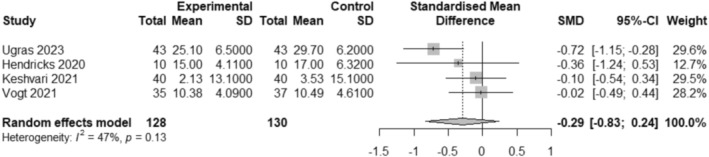
Forest plot of VR interventions compared with the control group for perioperative anxiety in adults after Leave‐One‐Out analysis.

**FIGURE 4:**
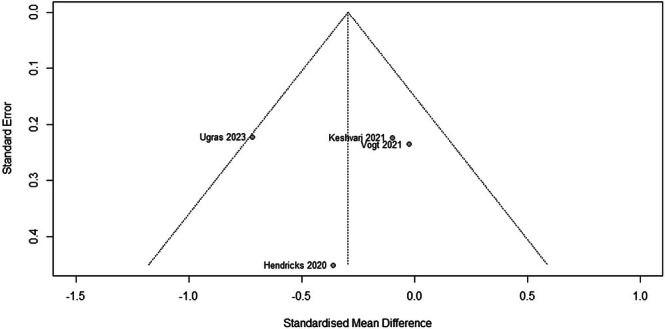
Funnel plot of VR interventions compared with the control group for perioperative anxiety in adults after Leave‐One‐Out analysis.

Leave‐one‐out analysis shows that heterogeneity is reduced to moderate (heterogeneity *I* ^2^ = 47%, *p* = 0.13) when excluding the Bekelis et al. (2017b) study. Details of the leave‐one‐out analysis are shown in Figure S1 (Baujat plot on influence and overall heterogeneity contribution) and Figure S2 (Effect size plot sorted by *I* ^2^ of full Leave‐one‐out analysis). The meta‐analysis of the remaining four studies (258 participants) is shown in Figure 3. Although favouring VR, the findings suggest a small and non‐significant reduction in preoperative anxiety (SMD −0.29, 95% CI −0.83 to 0.24). Inspection of the funnel plot (Figure 4) for the model shown in Figure 3 showed that the studies were symmetrically distributed around the pooled effect size, indicating a low risk of publication bias.

Three remaining RCTs were not included in the analysis for various reasons. Firstly, the study by Yang et al. (2019) was excluded due to skewed data distribution observed in the intervention group and thus the mean could not be calculated reliably from the median and IQR. Secondly, Robertson et al. (2017) utilised a summary measure of anxiety, specifically a change score rather than a mean score, which made it incompatible for inclusion. Lastly, the study by (Turrado et al. 2021) received a low‐quality score and presented inadequate analytical data, leading to its exclusion from the analysis. Moreover, no sub‐group analysis was done due to insufficient data.

#### 3.3.2 Secondary Outcomes

##### 3.3.2.1 VR Adverse Effects

The adverse effects of VR were evaluated in two studies (Baytar and Bollucuoğlu 2021; Turrado et al. 2021), but no specific assessment tool was used. In Baytar and Bollucuoğlu (2021), the VR group consisted of 58 participants who were asked to report any instances of headache, dizziness, or nausea. Similarly, Turrado et al. (2021) recruited 40 participants who were asked to report any adverse effects experienced in general. The results showed that only two participants, one from each study, reported mild dizziness. The duration of the VR session in both studies was comparable, which was 15 min in Baytar and Bollucuoğlu (2021) and 16 min and 34 s in Turrado et al. (2021).

##### 3.3.2.2 Stress Level

The effects of VR on stress levels were evaluated in two studies using 100‐point VAS scores, and their findings varied (Bekelis et al. 2017a; Yang et al. 2019). Bekelis et al. (2017b) reported a significant difference in preoperative stress levels between groups (difference:—41.7; 95% CI: −33.1 to −50.2; *p* < 0.01). In contrast, Yang et al. (2019) did not find a significant difference in median preoperative stress scores (VR: 40, 20.0–65.0 vs. control: 50, 22.5–70.0; *p* = 0.237). However, they did observe a significant difference in median postoperative stress scores, with the VR group showing lower scores than the control group (VR: 15, 2.5–37.5 vs. control: 30, 30.0–50.0; *p* = 0.012).

##### 3.3.2.3 Preparedness Level

Two studies evaluated the effects of VR on preparedness for surgery using VAS scores, and their findings differed (Bekelis et al. 2017a; Yang et al. 2019). Bekelis et al. (2017a) reported a significant difference in preparedness levels, indicating that the VR group had higher scores compared to the control group (difference, 32.4; 95% CI, 24.9–39.8; *p* < 0.01). However, Yang et al. (2019) did not find a significant difference in median preparedness scores between the groups (VR: 90, 70–100 vs. control: 80, 70–90; *p* = 0.145).

##### 3.3.2.4 Postoperative Pain

Two studies, conducted by Bekelis et al. (2017a) and Yang et al. (2019), examined the effects of VR on postoperative pain using VAS (Bekelis et al. 2017a; Yang et al. 2019). Both studies reported no significant difference in pain scores between the control group and the VR group. In the Yang et al. (2019) study, the median pain scores were similar in both groups (VR: 30, 12.0–49.0 vs. control: 30, 12.0–48.5; *p* = 0.265). Similarly, Bekelis et al. (2017a) found no significant difference in pain scores between the two groups at 24 h after surgery (−1.3; 95% CI, −4.2 to 6.8; *p* = 0.99) and at 30 days after surgery (−1.3; 95% CI, −4.2 to 6.8; *p* = 0.92).

##### 3.3.2.5 Patient Satisfaction

Four studies examined the effects of VR on perioperative patient satisfaction and consistently showed the effectiveness of VR in enhancing satisfaction levels (Baytar and Bollucuoğlu 2021; Kapikiran et al. 2022; Yang et al. 2019). Different assessment tools were used, including VAS satisfaction (Bekelis et al. 2017a; Yang et al. 2019), the Evaluation du Vécu de I'Anesthesie Generale (EVAN‐G) satisfaction score (Bekelis et al. 2017a) and the Newcastle Satisfaction with Nursing Care Scale (NSCS) (Kapikiran et al. 2022).

Bekelis et al. (2017a) assessed satisfaction twice, preoperatively using only VAS and within 24 h postoperatively using both VAS and EVAN‐G. The results showed a significant increase in perioperative satisfaction for the VR group compared to the control group, with a higher EVAN‐G score (difference: 20.0; 95% CI: 16.6–23.3) and VAS score (difference: 33.2; 95% CI: 25.4–41.0) in favour of VR. Similarly, Yang et al. (2019) assessed satisfaction using VAS before and after surgery. Consistently, the VR group reported higher median satisfaction scores than the control group, both preoperatively (VR: 95; 90.0–100.0 vs. Control: 85;70.0–96.0, *p* = 0.016) and on the third day postoperatively (VR: 95; 90.0–100.0 vs. Control: 85; 70.0–97.5, *p* = 0.010). Moreover, Kapikiran et al. (2022) reported a statistically significant increase in the mean satisfaction score among the VR group, measured 36 h before surgery (pre‐test) and 72 h after surgery (postest) using NSCS (*p* = 0.000, *t* = 1.214). The study also reported a moderately negative correlation between anxiety and satisfaction (*r* = −0.613, *p* = 0.000). Moreover, in the study of Baytar and Bollucuoğlu (2021), the participants were asked to rate their satisfaction with the VR experience using a simple scale ranging from “very satisfied” to “unsatisfied.” The findings showed that 90% of the participants were satisfied with their VR experience and expressed a desire to use VR again in future surgeries.

##### 3.3.2.6 Length of Hospital Stay

Three studies examined the effects of VR on hospital stay (Hendricks et al. 2020; Kapikiran et al. 2022; Turrado et al. 2021). Kapikiran et al. (2022) found that VR significantly reduced the length of hospital stay in transplant surgery patients (*χ* ^2^ = 7.51, *p* = 0.000, *F* = 2.154). Conversely, Hendricks et al. (2020) reported no significant differences in hospital stay groups for cardiac surgery. Similarly, Turrado et al. (2021) reported no significant differences in length of hospital stay between the two groups, with median stay 3.1 days (95% CI, 2.3–4.8 days) for patients who underwent colorectal surgery.

##### 3.3.2.7 Other Outcomes

Two studies investigated the effects of VR on several clinically relevant outcomes, including preoperative anxiolytic administration usage, difficulty of intubation, post‐operative nausea (Hendricks et al. 2020) and rate of complications (Turrado et al. 2021). In the study by Hendricks et al. (2020), although there was a reduction in the usage of preoperative anxiolytic administration in the VR group compared to the control group, this difference did not reach statistical significance (*p* = 0.08). No significant differences were identified between the two groups regarding the difficulty of intubation and postoperative nausea. Turrado et al. (2021) reported that there were no significant differences in complication rates between groups (VR: 5.17% vs. Control:5.88%).

## 4 Discussion

This review aimed to evaluate the effectiveness of VR interventions in reducing perioperative anxiety among adult elective surgical patients. While studies have shown a positive impact on anxiety reduction and patient experience in paediatric patients (Simonetti et al. 2022), there is limited research on the effectiveness of VR in adults. Addressing this research gap is crucial because preoperative anxiety is highly prevalent in adults (Abate et al. 2020) and can result in significant postoperative complications (Celik and Edipoglu 2018; Sobol‐Kwapinska et al. 2016).

A total of eleven studies were deemed eligible for inclusion in this review, eight RCTs (Bekelis et al. 2017a, 2017b; Hendricks et al. 2020; Keshvari et al. 2021; Robertson et al. 2017; Turrado et al. 2021; Ugras et al. 2022; Vogt et al. 2021; Yang et al. 2019) and three quasi‐experimental studies (Baytar and Bollucuoğlu 2021; Ganry et al. 2018; Kapikiran et al. 2022). During the quality assessment process, one study (Turrado et al. 2021) was identified as low quality and subsequently excluded from the meta‐analysis.

The review findings suggest that VR interventions hold promise as a potential strategy for reducing perioperative anxiety among patients. The use of VR for both educational and distraction purposes showed positive results in alleviating anxiety. Most studies (9/11, 82%) reported a significant reduction in preoperative anxiety when VR was used. A meta‐analysis, which included four studies, was conducted as part of this review, revealing a small effect size in terms of reducing preoperative anxiety (SMD −0.29, 95% CI −0.83 to 0.24) based on the subjective measurements. However, the results did not reach statistical significance. This lack of statistical significance can be attributed to the substantial heterogeneity observed among the included studies, indicating variations in study design, participant characteristics, VR interventions and outcome measures. These differences may have contributed to the inconsistency in the findings and limited the ability to draw definitive conclusions. While there is an indication of a potential benefit of VR interventions in reducing perioperative anxiety, the variability in the findings emphasises the necessity for future research with standardised methodologies and larger sample sizes to provide more conclusive evidence.

Almost half of the included studies in this review examined various hemodynamic parameters to assess the impact of VR interventions. Consistently, heart rate showed significant alterations, indicating that VR had an effect on heart rate (Baytar and Bollucuoğlu 2021; Keshvari et al. 2021; Ugras et al. 2022). However, the effects on other hemodynamic parameters, such as blood pressure, respiratory rate and oxygen saturation, were either minimal or inconsistent across the studies. This suggests that VR interventions may not directly influence these specific hemodynamic parameters significantly in the perioperative care context. Regarding biochemical markers, the findings suggest that VR interventions may have a measurable impact on certain markers, such as galvanic skin response (Robertson et al. 2017) and salivary cortisol levels (Ganry et al. 2018), indicating a potential physiological response to VR.

Despite the limited changes in hemodynamic parameters, the participants reported high satisfaction with VR interventions. Their subjective experiences and perceptions of VR interventions were generally positive, as indicated by consistently high levels of reported satisfaction across the studies that assessed the satisfaction levels (Baytar and Bollucuoğlu 2021; Kapikiran et al. 2022; Yang et al. 2019). This highlights the value that participants found in using VR to reduce anxiety and enhance their overall perioperative experience, even if the objective changes in hemodynamic parameters were not significant.

### 4.1 Border Application of VR in Healthcare

The application of VR in healthcare settings, such as dentistry, oncology and general medical procedures, has gained attention. In dentistry, VR has emerged as a promising approach to address pain experienced by patients during dental treatments. A meta‐analysis incorporating ten studies, including eight involving paediatric and two adult patients, demonstrated that VR was an effective distraction technique, resulting in a significant reduction in pain levels (SMD −0.82, 95% CI −1.42 to −0.22) among paediatric patients. However, the findings did not reach statistical significance in the adult population (SMD −0.67, 95% CI −1.58 to 0.24) (López‐Valverde et al. 2020). Similarly, for anxiety experienced during dental treatments by both paediatric and adult patients, the results did not show statistical significance (López‐Valverde et al. 2020). In oncology, a systematic review and meta‐analysis of six studies demonstrated that immersive VR interventions are effective in reducing pain (SMD −1.08, 95% CI −1.65 to −0.51) and anxiety (SMD −1.86, 95% CI −2.98 to −0.73) among children and adolescents undergoing standard oncologic care procedures (Czech et al. 2023). In the field of medical procedures, VR has gained traction as a potential intervention as well. Smith et al. (2020) conducted an extensive systematic review involving 18 studies that focused on the use of VR as an analgesic and anxiolytic tool in an inpatient setting, including both children and adults. The findings from the review revealed that a significant 67% of the studies reported reductions in pain when VR was used, indicating its efficacy as a pain management strategy. Moreover, 50% of the studies highlighted significant reductions in procedural anxiety, underscoring the potential of VR in alleviating anxiety during medical procedures (Smith et al. 2020).

### 4.2 Safety and Adverse Effects VR

This review found that the adverse effects of VR interventions in perioperative care were generally minimal, with only a few participants reporting mild dizziness. This aligns with previous studies conducted with paediatric and adolescent patients, which also reported few side effects such as motion sickness or fatigue associated with the use of VR (Dascal et al. 2017; Ridout et al. 2021). However, it is important to note that the assessment of adverse effects specifically related to VR interventions was limited in the included studies, with only two studies addressing this aspect. Furthermore, the tools utilised for evaluating adverse effects were not explicitly specified or validated. Hence, there is a need for more comprehensive and standardised assessment methods to accurately evaluate and monitor the potential adverse effects of VR interventions in perioperative care.

### 4.3 Limitations and Future Directions

The overall completeness and applicability of evidence regarding the effectiveness of VR in reducing perioperative anxiety in adult surgical patients may have some limitations. Firstly, the number of available studies on this specific topic may be limited, which could affect the comprehensiveness of the evidence base. Additionally, there are variations in study design, sample size, patient characteristics, VR interventions and outcome measures among the included studies, which could limit the generalisability of the findings. Moreover, the majority of the studies focused on elective surgical patients, and the results may not be applicable to patients undergoing emergency or high‐risk surgeries. The duration and timing of the VR interventions also varied across studies, and the optimal timing and duration for maximum effectiveness are yet to be determined. Furthermore, most studies primarily relied on self‐reported measures of anxiety, and objective measurements were limited. In addition, all surgical patients were included regardless of their anxiety levels, with only one quasi‐experimental study screening patients based on moderate to high levels of preoperative anxiety for the VR session (Ganry et al. 2018). Screening patients for their anxiety levels before including them in the VR intervention helps ensure that the study focuses on individuals who may benefit the most from anxiety reduction strategies. By including patients with moderate to high levels of preoperative anxiety, the effectiveness of VR interventions can be evaluated in a targeted sub‐group. This approach allows for a more precise assessment of VR's impact on anxiety levels and provides insights into its benefits for those with higher anxiety levels. Additionally, screening helps ensure appropriate support is provided to those who may require additional assistance in managing their anxiety during the perioperative period.

The study overview reveals several potential biases that need to be considered. Three out of the eight included RCTs had unclear allocation concealment, raising concerns about selection bias. Furthermore, due to the nature of the VR intervention, blinding of participants was not feasible, introducing the potential for performance bias. Participants' expectations and beliefs about the intervention could influence their reported outcomes. However, implementing blinding procedures for outcome assessors and data analysts can still help reduce detection bias. Nevertheless, half of the included studies did not provide clear information about whether the outcome assessors were blinded to treatment assignment, introducing the risk of bias in outcome assessment. Clear reporting and implementation of blinding procedures for outcome assessors are crucial to minimise bias and maintain the objectivity of outcome measurements in future studies.

## 5 Conclusion

The reviewed studies present a contrasting body of evidence regarding the effectiveness of VR in reducing preoperative anxiety and enhancing patient satisfaction in surgical settings. While 80% of studies demonstrated significant results, others failed to find significant effects. These divergent findings, coupled with the heterogeneity among the studies, make it challenging to draw a clear and definitive conclusion. The limitations observed in the reviewed studies, such as small sample sizes and the lack of standardised measures for anxiety assessment, highlight the need for more well‐designed research in this area. Future studies should consider incorporating larger sample sizes and utilising standardised measures to ensure consistency and comparability across studies. Furthermore, it is essential to address the variations in VR interventions and the duration of exposure to determine optimal protocols for anxiety reduction. Despite the limitations, the potential benefits of VR in surgical settings cannot be dismissed. The positive findings regarding reduced preoperative anxiety and increased patient satisfaction provide valuable insights. However, caution should be exercised in interpreting these results due to the limitations and inconsistencies observed. To provide more robust evidence on the effectiveness of VR, researchers, healthcare professionals and technology developers should collaborate to conduct rigorous studies that adhere to standardised methodologies. By addressing the existing limitations and building upon the promising findings, a clearer understanding of the role of VR in reducing perioperative anxiety and enhancing patient satisfaction can be achieved.

## Author Contributions

**Salihah Asiri:** conceptualisation, search strategy, study selection, data extraction, quality assessment, data synthesis, writing – original draft, and writing – review and editing. **Jane Currie:** data synthesis and writing – review and editing. **Jed Duff:** conceptualisation, study selection, data synthesis, and writing – review and editing. **Michelle Guilhermino:** conceptualisation, data extraction, quality assessment, data synthesis, interpretation of result, and writing – review and editing.

## Conflicts of Interest

The authors declare no conflicts of interest.

## Supporting information


Appendix S1.

Appendix S2.

Figures S1–S2.

## Data Availability

The data are AVAILABLE ON REQUEst from the corresponding author.
