# Supplementary material for: Effectiveness of Virtual Reality Interventions for Perioperative Anxiety in Adults: A Systemic Review With Meta‐Analysis
Source: J Clin Nurs. 2025 May 23;34(9):3539–59. doi: 10.1111/jocn.17806 (PMC12340745; doi:10.1111/jocn.17806)
Supplement: Supplementary file 2 — Appendix S2. [file JOCN-34-3539-s003.docx]

JBI Critical Appraisal Checklist for
quasi-experimental studies

Reviewer ______________________________________ Date_______________________________

Author_______________________________________ Year_________ Record Number_________

|  | Yes | No | Unclear | Not applicable |
| --- | --- | --- | --- | --- |
| 1. Is it clear in the study what is the ‘cause’ and what is the ‘effect’ (i.e. there is no confusion about which variable comes first)? | □ | □ | □ | □ |
| 1. Were the participants included in any comparisons similar? | □ | □ | □ | □ |
| 1. Were the participants included in any comparisons receiving similar treatment/care, other than the exposure or intervention of interest? | □ | □ | □ | □ |
| 1. Was there a control group? | □ | □ | □ | □ |
| 1. Were there multiple measurements of the outcome both pre and post the intervention/exposure? | □ | □ | □ | □ |
| 1. Was follow up complete and if not, were differences between groups in terms of their follow up adequately described and analyzed? | □ | □ | □ | □ |
| 1. Were the outcomes of participants included in any comparisons measured in the same way? | □ | □ | □ | □ |
| 1. Were outcomes measured in a reliable way? | □ | □ | □ | □ |
| 1. Was appropriate statistical analysis used? | □ | □ | □ | □ |

Overall appraisal: Include □ Exclude □ Seek further info □

Comments (Including reason for exclusion)

____________________________________________________________________________________________________________________________________________________________________________________
