# Supplementary material for: Effectiveness of Virtual Reality Interventions for Perioperative Anxiety in Adults: A Systemic Review With Meta‐Analysis
Source: J Clin Nurs. 2025 May 23;34(9):3539–59. doi: 10.1111/jocn.17806 (PMC12340745; doi:10.1111/jocn.17806)

**Supplementary Figure 1.**Baujat plot used to detect and diagnose which study is overly contributing to heterogeneity in this meta analysis, in this case the Bekelis 2017 study. The overall heterogeneity is on the horizontal axis with each studies influence on the pooled effect size on the vertical axis.


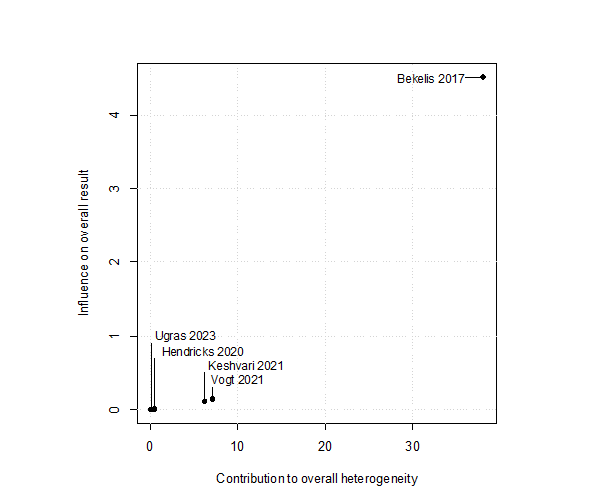


**Supplementary Figure 2.**Full leave-one-out analysis showing effect size (Random-Effects Model) sorted by I^2^ when each study was omitted from the analysis. Shaded area is the original 95% confidence interval of the original pooled effect size and the dashed line is the original pooled effect size estimate. Heterogeneity is reduced to moderate levels (47%) only when omitting Bekelis 2017.


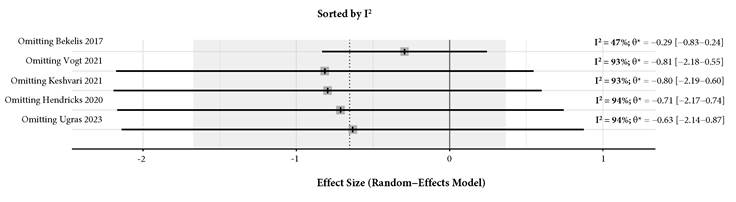

Supplement: Supplementary file 3 — Figures S1–S2. [file JOCN-34-3539-s001.docx]
